# From Commensal to Pathogen: Unveiling Klebsiella pneumoniae subsp. ozaenae as a Cause of Meningitis and a Literature Review

**DOI:** 10.7759/cureus.96255

**Published:** 2025-11-06

**Authors:** Premnath CS, Vasundhara Devi P, Sreenivasulu Reddy Palukuru, Chaitanya Jakkam

**Affiliations:** 1 Microbiology, Narayana Medical College and Hospital, Nellore, IND; 2 Critical Care Medicine, Narayana Medical College and Hospital, Nellore, IND

**Keywords:** bacterial meningitis, case report, immunocompromised host, klebsiella ozaenae, rare infection, vitek 2

## Abstract

*Klebsiella pneumoniae *subsp.* ozaenae* is a very rare pathogen; in the majority of cases, it is a commensal in the upper respiratory tract, causing chronic atrophic rhinitis; however, it is now recognized as a cause of many invasive infections. We report a rare case of meningitis in a young man with a history of uncontrolled diabetes mellitus and hypertension, where *Klebsiella ozaenae* isolation from cerebrospinal fluid made the diagnosis definite, and also a literature review. In spite of the treatment received with ceftriaxone and vancomycin, he unfortunately succumbed to the disease. After a thorough literature review, this is the first fatal documented case of *Klebsiella ozaenae *meningitis in a young male in India that proves the rarity of this infection as well as the virulence of the organism.

## Introduction

*Klebsiella pneumoniae* subsp. *ozaenae* meningitis is a highly unusual clinical condition and frequently goes unnoticed due to its relation to chronic nasal colonization and atrophic rhinitis, in contrast to invasive infection [1]. The organism, once regarded as an upper respiratory tract commensal, has more and more been found to cause severe diseases like bacteremia, pneumonia, meningitis, and endogenous endophthalmitis, especially in the elderly or immunocompromised host [2-4]. Multiple studies have identified chronic rhinitis, older age, prior antibiotic therapy, immunosuppressive states, malignancy, alcohol use, and diabetes mellitus as key predisposing factors for *Klebsiella ozaenae* infections, including bacteremia [5].

Its unfamiliarity, unremarkable clinical course, and lack of similarity to more usual central nervous system bacterial infections frequently result in delayed diagnosis or misdiagnosis.

We present the case of *Klebsiella ozaenae* meningoencephalitis in an uncontrolled diabetic, a chronic alcoholic, and a hypertensive young male, and a literature review.

## Case presentation

A 37-year-old adult male patient presented to the emergency department with complaints of high-grade fever for one week associated with headache, vomiting, and altered sensorium for three days, and then became irritable and not oriented to time, place, or person. There was no history of trauma and seizures. Prior history of immunosuppression with a history of recurrent skin and soft tissue infections due to uncontrolled type 1 diabetes mellitus for 10 years, and also a known hypertensive for five years, a chronic alcoholic with a history of acute pancreatitis one year back.

Vitals included a pulse of 90/minute in sinus rhythm and blood pressure of 160/70 mmHg.

On examination, the patient was not responsive to verbal or painful stimuli, with a Glasgow Coma Scale (GCS) score of E1V2M4 = 7/15, indicating a severe impairment of consciousness. Cranial nerve power was normal. Power: upper limbs 1/5, lower limbs 2/5 bilaterally. Deep tendon reflexes: upper and lower limbs are both present. Plantar reflex: Bilateral flexor, neck rigidity, with the Kernig sign positive.

Meningitis was clinically suspected based on signs and symptoms and subsequently confirmed by cerebrospinal fluid (CSF) examination and lumbar puncture, and blood cultures were obtained prior to initiating empiric antimicrobial therapy. Empiric therapy with intravenous ceftriaxone 2 g every 12 hours and vancomycin 1.25 g every 24 hours was begun shortly after lumbar puncture and was given following a course of dexamethasone 10 mg every six hours before antibiotics were started.

Blood tests showed hemoglobin of 12.7 g/dL; the leukocyte count was 39,900 cells/μL with 94% neutrophils, herein reflective of an inflammatory response directed toward bacterial infection, and platelets were also elevated to 5.15 lakh/μL, suggestive of reactive thrombocytosis, as shown in Table 1. Serum electrolytes were within normal limits, and urine culture did not yield any growth after 24 hours of incubation.

**Table 1:** Laboratory and cerebrospinal fluid (CSF) findings on admission. ADA = adenosine deaminase; LDH = lactate dehydrogenase

Laboratory Parameter	Result	Reference Range
Hemoglobin (g/dL)	12.7	13.6-17.2 (males)
Total leukocyte count (cells/µL)	39,900	4,000-11,000
Neutrophils (%)	94	40-75
Lymphocytes (%)	2	20-40
Monocytes (%)	3	2-8
Eosinophils (%)	1	1-6
Basophils (%)	0	0-1
Platelet count (/µL)	515,000	150,000-400,000
RBC count (million/µL)	3.2	4.3-5.9 (males)
Packed cell volume (%)	37	40-54 (males)
Blood urea (mg/dL)	40.02	17-43
Serum creatinine (mg/dL)	1.29	0.7-1.3
C-reactive protein (mg/L)	155	5-500
CSF total cells (cells/µL)	5,000	< 5
CSF neutrophils (%)	95	0-6
CSF lymphocytes (%)	5	40-80
CSF glucose (mg/dL)	25	45-80
CSF protein (mg/dL)	310	15-45
CSF LDH (U/L)	15,593	10-30
CSF ADA (U/L)	12	< 10

**Figure 1:**
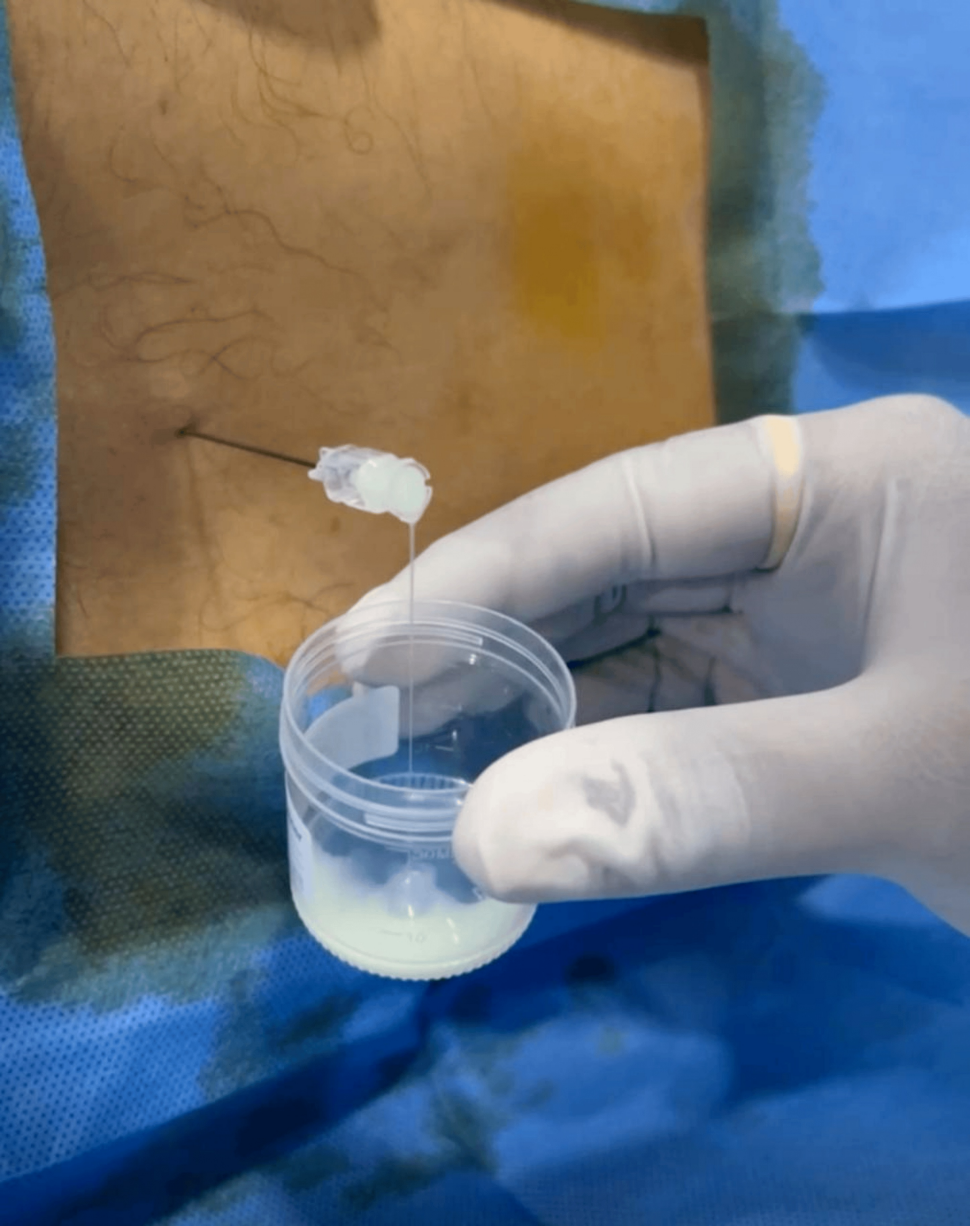
Gross appearance of cerebrospinal fluid (CSF) sample.

The CSF examination sample appeared milky white and cloudy on gross inspection, as shown in Figure 1. Microscopy revealed a total of 5,000 cells/μL with 95% neutrophils and 5% lymphocytes. Biochemistry tests were in favor of bacterial meningitis and revealed a low glucose level (25 mg/dL), a highly elevated protein level (310 mg/dL), and a very high lactate dehydrogenase (LDH) of 15,593 U/L. ADA was 12 U/L, which did not indicate tuberculous involvement, as shown in Table 1.

Other infections, hepatitis B, hepatitis C, HIV, leptospirosis, malaria, and scrub typhus, were all negative by rapid immunochromatographic tests.

**Figure 2:**
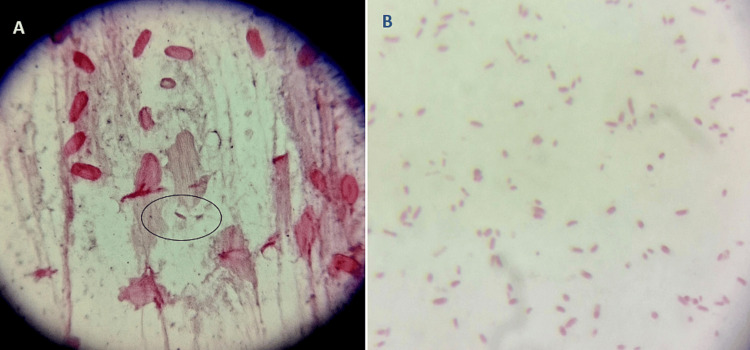
(A) Gram-negative bacilli (shown in the circle) with abundant pus cells on Gram stain of cerebrospinal fluid (CSF) (B) Colony smear showing short and stout Gram-negative bacilli under 100× oil immersion objective.

Microbiological analysis further supported the diagnosis. Gram stain of the CSF demonstrated Gram-negative bacilli with abundant pus cells and short and stout Gram-negative bacilli from the culture smear, as shown in Figure 2.

**Figure 3:**
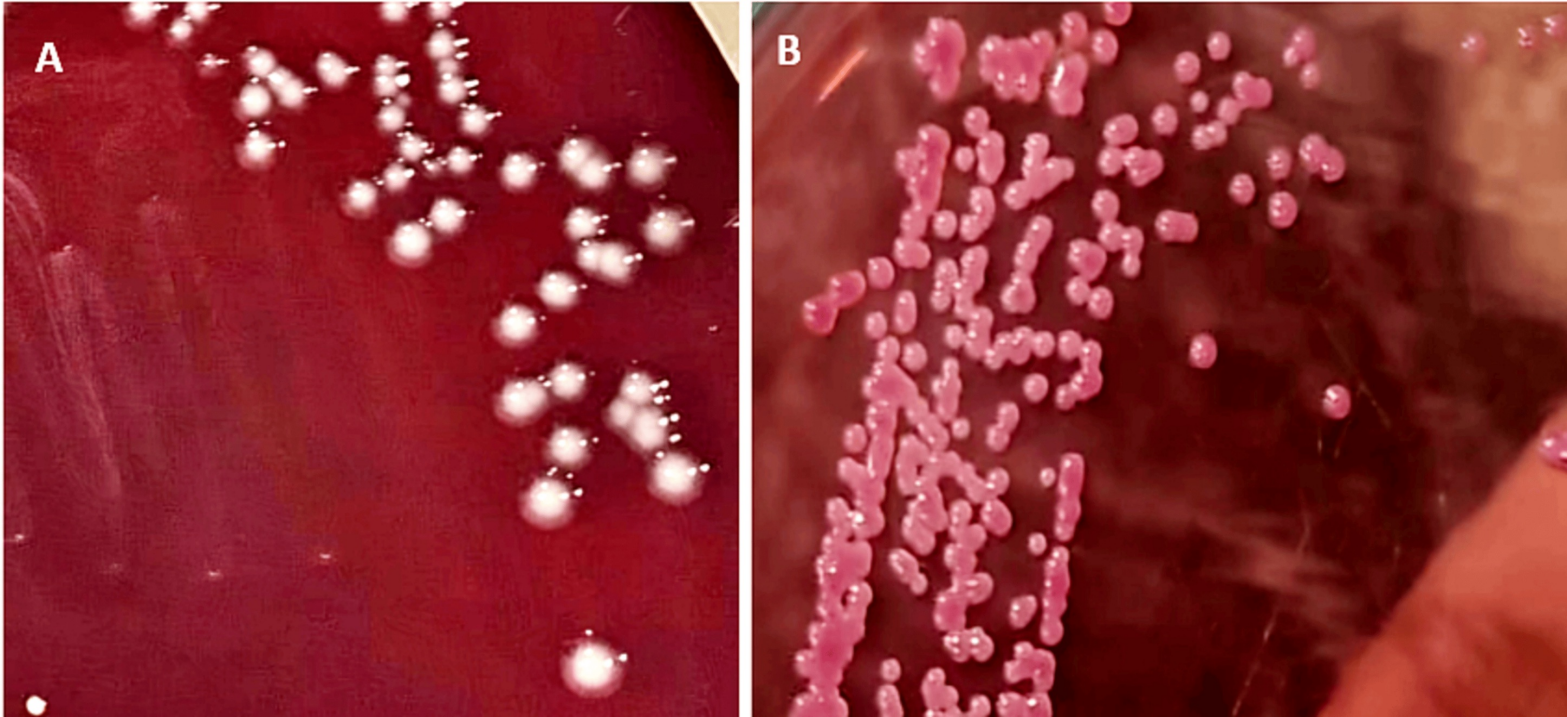
(A) Growth on blood agar showing greyish, smooth, non-hemolytic colonies (B) Lactose-fermenting, smooth, pink mucoid colonies on MacConkey agar of Klebsiella pneumoniae subsp. ozaenae.

In CSF and blood cultures, large mucoid, pink lactose-fermenting colonies grew on MacConkey agar, while blood agar showed large greyish, non-hemolytic colonies (Figure 3).

Biochemical findings include no indole produced, citrate not utilized, urease not hydrolyzed, and TSI acid slant and butt with no H₂S production.

The isolate was finally identified as *Klebsiella pneumoniae* subsp. *ozaenae* using the VITEK® 2 Compact system (BioMérieux, Marcy-l’Étoile, France) with 95% confidence, and the drug susceptibility results are presented in Table 2. This confirmed an unusual and rare cause of bacterial meningitis in this patient.

**Table 2:** Antimicrobial susceptibility results of Klebsiella pneumoniae subsp. ozaenae as determined by the VITEK® 2 Compact system (bioMérieux, Marcy-l’Étoile, France). I = intermediate; MIC = minimum inhibitory concentration; R = resistant; S = susceptible

Antimicrobial	MIC (µg/mL)	Interpretation
Amoxicillin/clavulanic acid	≤2	S
Piperacillin/tazobactam	≤4	S
Cefuroxime	2	S
Cefuroxime axetil	2	S
Ceftriaxone	≤0.25	S
Cefoperazone/sulbactam	≤8	S
Cefepime	≤0.12	S
Ertapenem	≤0.12	S
Imipenem	≤0.25	S
Meropenem	0.25	S
Amikacin	≤1	S
Gentamicin	≤1	S
Ciprofloxacin	≤0.06	S
Colistin	2	I
Trimethoprim/sulfamethoxazole	≥320	R

**Figure 4:**
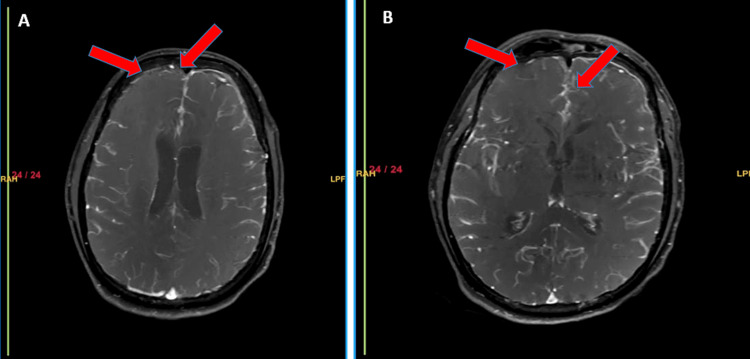
(A) Axial T1-weighted image with contrast (T1+C) showing leptomeningeal enhancement along the bilateral frontoparietotemporal convexities (red arrows). (B) Axial T1+C at a lower level showing pachymeningeal and tentorial enhancement (red arrows).

**Figure 5:**
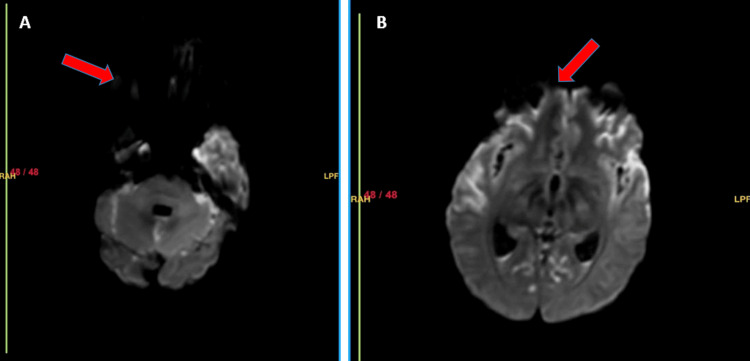
(A) Axial diffusion weighted imaging (DWI) showing gyriform and sulcal hyperintensity in the bilateral frontoparietotemporal and posterior fossa sulci (red arrow) consistent with diffusion restriction. (B) Corresponding apparent diffusion coefficient (ADC) map demonstrating low ADC in the same sulci (red arrow), confirming true diffusion restriction.

Magnetic resonance imaging (MRI) of the brain with contrast demonstrated findings consistent with bacterial meningitis with extensive meningeal involvement. Gyriform areas of FLAIR hyperintensity with true diffusion restriction and post-contrast enhancement were seen involving the bilateral fronto-parieto-temporal sulci, tentorium, cerebellum, and vermis (Figures 4-5). There was also leptomeningeal and pachymeningeal thickening with enhancement along the left frontal convexity, bilateral tentorium, and cerebellopontine angles. The basal ganglia, thalami, paranasal sinuses, and mastoid air cells appeared unremarkable.

Course of disease and outcome

In spite of broad-spectrum antibiotics, the condition of the patient kept deteriorating. He had progressive neurological deterioration, with rising confusion, unremitting high fever, and labile blood pressure. Even inflammatory markers failed to normalize, and metabolic acidosis worsened. The clinical course was also further complicated by diffuse brain edema, and the patient developed refractory septic shock unresponsive to vasopressors. The patient died on day 6 of admission. The immediate cause of death was fulminant Gram-negative bacterial meningitis caused by *Klebsiella ozaenae* with cerebral edema and septic shock as complicating factors.

## Discussion

Literature review

*Klebsiella ozaenae* meningitis is very rare, and only a few cases have been reported in the English literature. Lewis et al. [6], in 1979, reported the first case in a 62-year-old man with pneumonia and hyperglycemia. Both blood cultures and CSF grew *Klebsiella ozaenae*, but the patient died within 48 hours of penicillin, chloramphenicol, and gentamicin therapy.

Later, Siegel, in 1987 [7], documented a 78-year-old diabetic woman with diabetes mellitus, chronic sinusitis, and otitis media whose CSF, middle ear, and maxillary sinus cultured *Klebsiella ozaenae*. She recovered following cefotaxime therapy.

Further reports from Asia broadened the understanding of this pathogen. Tang and Chen [2], 1994, reported two more cases of meningitis due to *Klebsiella ozaenae*, one in the context of nasopharyngeal carcinoma, both of which were treated successfully with proper antimicrobial chemotherapy. Likewise, Pusponegoro et al., 1998 [8], in their epidemiologic study from Jakarta and Tangerang, Indonesia, identified *Klebsiella* species among the etiologic agents of bacterial meningitis, although *Klebsiella ozaenae* remained an uncommon bacterium.

A new Japanese report of *Klebsiella ozaenae* meningitis by Hosono Honda et al., 2024 [9], in an uncontrolled diabetic woman in her 70s with frontal sinusitis included initial matrix-assisted laser desorption/ionization time-of-flight (MALDI-TOF) misidentification as *Klebsiella pneumoniae* and subsequent identification by whole genome sequencing as *Klebsiella ozaenae* (K4-ST91); she resolved with ceftriaxone followed by empiric meropenem/vancomycin for a course.

A summary of previously reported cases is presented in Table 3 [2,6-9].

**Table 3:** Summary of previously reported cases of meningitis by Klebsiella ozaenae.

Author (Year)	Patient Age	Sex	Associated Condition(s)	Culture Source	Antibiotics Used	Outcome
Tang and Chen, 1994 [2]	55, 53	M, F	Nasopharyngeal carcinoma; atrophic rhinitis, turbinectomy, ethmoidectomy	CSF	Penicillin-based cephalosporins	Recovery
Lewis et al., 1979 [6]	62	M	Hyperglycemia, pneumonia	CSF, blood	Penicillin, chloramphenicol, gentamicin	Death
Siegel, 1987 [7]	78	F	Diabetes mellitus, chronic sinusitis, otitis media	CSF, middle ear, maxillary sinus	Cefotaxime	Recovery
Pusponegoro et al., 1998 [8]	-	-	Epidemiologic study, Jakarta/Tangerang: *Klebsiella* spp. meningitis (one isolate of *Klebsiella ozaenae* out of 11 cases)	CSF	Ampicillin and chloramphenicol	Recovery
Hosono Honda et al., 2024 [9]	70s	F	Uncontrolled diabetes mellitus, frontal sinusitis	CSF	Meropenem + vancomycin (empiric), then ceftriaxone	Recovery

*Klebsiella pneumoniae* subsp. *ozaenae* is an extremely rare cause of meningitis, with only a handful of reported cases globally since 2000 [9]. It is usually found in diabetic or elderly patients and can be secondary to infection of the sinonasal [5-7]. The case described here, as detected by VITEK 2, depicts the invasive nature of the pathogen even in the absence of overt sinus disease and also the first fatal instance of *Klebsiella ozaenae* meningitis in a young male who was a poorly controlled diabetic and chronic alcoholic from India.

The CSF profile of neutrophilic pleocytosis, very elevated protein and LDH, and low glucose had been linked with bacterial meningitis of Gram-negative etiology. Mucoid, lactose-fermenting colonies on MacConkey agar and Gram-negative bacilli on smear established the diagnosis. Dexamethasone, ceftriaxone, and vancomycin given early were not enough; the patient progressed rapidly and died of cerebral edema and septic shock.

In contrast with the Japanese case that was reported by Hosono Honda et al. in 2024 [9] and was well-recovered on ceftriaxone therapy, this result suggests the fulminant and unpredictable character of *Klebsiella ozaenae* meningitis. Automated systems such as VITEK 2 provide good identification and direct therapy, although genomic validation is still the ultimate diagnostic gold standard.

Though *Klebsiella pneumoniae* and *Klebsiella ozaenae* can be distinguished biochemically by means of the negative Voges-Proskauer test and malonic acid utilization, discrimination by MALDI-TOF mass spectrometry is hampered by overlapping spectral profiles, and thus *Klebsiella ozaenae* has often been misidentified as *Klebsiella pneumoniae*. MALDI-TOF is fast for genus-level identification but lacks resolution for subspecies discrimination due to a limited database [9]. In contrast, whole-genome sequencing (WGS) gives unambiguous identification at the species and subspecies level when average nucleotide identity (ANI) is compared; a strain identified as *Klebsiella ozaenae* shared 99.76% ANI with capsule type K4 and sequence type ST91 [9]. WGS also conveys full information on virulence and resistance genes; however, its use in routine diagnostics is restricted due to costs and processing time.

Limitations

Genomic sequencing and molecular resistance testing were not undertaken, and since this is a single case only, the result cannot be extrapolated. However, the inclusion of this case does provide important information regarding the clinical spectrum and outcome of this unusual infection.

## Conclusions

*Klebsiella pneumoniae* subsp. *ozaenae* is an extremely infrequent etiology of bacterial meningitis. The case report, as entered by VITEK 2 Compact, points to the value of automated identification systems and diligent biochemical/CSF correlation. *Klebsiella ozaenae* meningitis has been described in the literature as having high morbidity. Clinician-microbiologist vigilance is paramount for timely diagnosis, vigorous supportive care, and keeping uncommon pathogens in mind in incomprehensible cases.

The limited discriminatory power of MALDI-TOF MS necessitates confirmatory techniques, such as WGS, for accurate species and subspecies identification, which are crucial not only for treatment decisions but also for epidemiological tracking. Rapid progression to cerebral edema and septic shock in this case also highlights the fulminant nature of *Klebsiella ozaenae* meningitis and the potential for poor outcomes despite early broad-spectrum antibiotic therapy.
